# Metabolomic Profiling of Asparagine Deprivation in Asparagine Synthetase Deficiency Patient-Derived Cells

**DOI:** 10.3390/nu15081938

**Published:** 2023-04-18

**Authors:** Mario C. Chang, Stephen J. Staklinski, Vinay R. Malut, Geraldine L. Pierre, Michael S. Kilberg, Matthew E. Merritt

**Affiliations:** 1 Department of Biochemistry and Molecular Biology, College of Medicine, University of Florida, Gainesville, FL 32610, USA; 2 School of Biological Sciences, Cold Spring Harbor Laboratory, Cold Spring Harbor, NY 11724, USA

**Keywords:** GC-MS, metabolomics, amino acids, asparagine, deprivation, ASNSD, biogenic, cancer metabolism, high-throughput, translational

## Abstract

The natural amino acid asparagine (Asn) is required by cells to sustain function and proliferation. Healthy cells can synthesize Asn through asparagine synthetase (ASNS) activity, whereas specific cancer and genetically diseased cells are forced to obtain asparagine from the extracellular environment. ASNS catalyzes the ATP-dependent synthesis of Asn from aspartate by consuming glutamine as a nitrogen source. Asparagine Synthetase Deficiency (ASNSD) is a disease that results from biallelic mutations in the ASNS gene and presents with congenital microcephaly, intractable seizures, and progressive brain atrophy. ASNSD often leads to premature death. Although clinical and cellular studies have reported that Asn deprivation contributes to the disease symptoms, the global metabolic effects of Asn deprivation on ASNSD-derived cells have not been studied. We analyzed two previously characterized cell culture models, lymphoblastoids and fibroblasts, each carrying unique ASNS mutations from families with ASNSD. Metabolomics analysis demonstrated that Asn deprivation in ASNS-deficient cells led to disruptions across a wide range of metabolites. Moreover, we observed significant decrements in TCA cycle intermediates and anaplerotic substrates in ASNS-deficient cells challenged with Asn deprivation. We have identified pantothenate, phenylalanine, and aspartate as possible biomarkers of Asn deprivation in normal and ASNSD-derived cells. This work implies the possibility of a novel ASNSD diagnostic via targeted biomarker analysis of a blood draw.

## 1. Introduction

Asparagine (Asn) is a biogenic amino acid required by all cells for proper growth and function. Healthy cells predominantly attain Asn through de novo biosynthesis. Asparagine Synthetase (ASNS) is the only human enzyme that catalyzes the biosynthesis of Asn, converting aspartate to Asn using glutamine as the nitrogen donor, in an ATP-dependent manner [1,2,3]. The human *ASNS* gene occupies 35 kb within chromosome 7q21.3 and is composed of a total of 13 exons [4,5,6]. Translation of the ASNS mRNA expresses an active enzyme (~64 kDa) consisting of 561 amino acids and two distinct functional domains [1,7]. *ASNS* transcription is heavily regulated by cellular perturbations such as endoplasmic reticulum stress or amino acid deprivation [1]. Structural modeling observed significant similarity between human ASNS and *E. coli* AS-B proteins and led to a proposed mechanism that begins with glutaminase activity in the *N*-terminal domain, which generates glutamate and a free ammonia molecule that passes through an intermolecular tunnel to the *C*-terminal domain [2,7,8,9]. ATP and aspartate bind within the *C*-terminal domain to form a β-aspartyl-AMP intermediate that is attacked by the free ammonia group and thus forms Asn.

Recently, ASNS expression and activity have been implicated in the onset and progression of specific diseases. ASNS acts as a strong promoting factor of cell proliferation and metastasis across a variety of different cancers [3]. Several studies have found that Asn can rescue and sustain cell proliferation and viability through protein synthesis, as an amino acid exchange factor, and by coupling mitochondrial respiration to cell growth [10,11,12]. Interestingly, in one instance, the significance of Asn for cancer cell proliferation was initially identified by the anti-cancer efficacy of extracellular asparaginase treatment of childhood acute lymphoblastic leukemia (ALL), which typically expresses low levels of ASNS [13,14,15,16]. Another disease associated with Asn bioavailability is asparagine synthetase deficiency (ASNSD), which was first described in 2013 as an inborn error of metabolism caused by deleterious biallelic mutations in the *ASNS* gene [17]. As of now, more than 75 ASNSD patients across 55 different families have been reported [1,4,17,18,19,20,21]. The explicit molecular mechanisms behind the clinical symptoms of the disease are not fully elucidated, and clinical assessment of children with ASNSD has revealed severe developmental delay resulting in congenital microcephaly, intractable seizures, hypotonia, appendicular spasticity, and brain atrophy, all which often lead to the premature death of the children affected.

Characterizations of ASNSD patient-derived cell lines in culture have revealed that, in the absences of medium-supplied Asn, the ASNSD cells exhibit suppressed cellular proliferation that usually correlates with the enzymatic activity of the variant protein [20,22,23,24,25,26]. However, a global metabolomics analysis exploring the effect of Asn deprivation has yet to be performed on ASNSD cells. To have a greater understanding of the cellular consequences of Asn deprivation, it is important to elucidate the metabolic changes occurring in Asn-deprived cells. ASNS is intimately connected to the tricarboxylic acid (TCA) cycle, glycolysis, amino acid metabolism, and energy metabolism through the consumption of aspartate, glutamine, and ATP. Metabolic stress caused by Asn deprivation in ASNS-deficient cells can reveal the role ASNS plays in global metabolism. Metabolomics, the global analysis of metabolites across all cellular pathways, can systematically detect metabolic changes. Gas chromatography-mass spectrometry (GC-MS) has been extensively used for the investigation of biomarkers [27] because there are well-established methods for a broad scope of metabolites including amino acids, carbohydrates, central carbon intermediates, and lipids [27,28].

We hypothesized that Asn deprivation would significantly change the global metabolic profile of ASNS-deficient cells, and global metabolomics would reveal the effects of Asn deprivation on ASNSD-derived lymphoblastoids and fibroblasts and possibly identify potential biomarkers of Asn deprivation. We report a GC-MS metabolomics panel and multivariate statistical analysis assessing changes in the global metabolic profiles of patient-derived cells challenged with Asn deprivation. The present results uncover a significant decrement in central carbon, amino acid, and anaplerotic metabolism with a particular insult to the TCA cycle of cells with deficient ASNS activity.

## 2. Materials and Methods

### 2.1. Reagents and Cell Lines

Patient-derived lymphoblastoid cell lines (LCL) were generated from an ASNSD-derived compound heterozygotic child (p.H205P and p.Y398Lfs*4 ASNS variants) as well as the child’s mother (p.H205P) and father (p.Y398Lfs*4), as previously reported in [25]. An unrelated wild-type EBV-immortalized human B-lymphoblastoid cell line was purchased from Cellero (1038-2750JA15, Memphis, TN, USA). Patient fibroblast cell lines were derived from skin biopsies taken from an ASNSD-affected child (G373V and R519H ASNS variants) as well as the mother (R519H) and father (G373V), as reported previously [22]. An unrelated wild-type (WT) adult dermal fibroblast cell line was purchased from American Type Culture Collection (PCS-201-012, Manassas, VA, USA). Roswell Park Memorial Institute Medium (RPMI 1640) with Asn (10-040-CV) or without Asn (custom formulation), Dulbecco’s Modified Eagle’s Medium (DMEM, 10-013-CV), 1X Penicillin/Streptomycin (P/S, 30-002-CI), ABAM (streptomycin, penicillin G, and amphotericin B, 30-004-CI), 1X non-essential amino acids (NEAA, 25-025-CL), and L-Glutamine (Gln, 25-005-Cl) were obtained from Corning (Corning, NY, USA). Dialyzed fetal bovine serum (dFBS, S12850) used for experimental media and fetal bovine serum (FBS, S11550) used for cell maintenance were acquired from Biotechne (Minneapolis, MN, USA). Acetonitrile, isopropanol, as well as methoxyamine hydrochloride dissolved in pyridine (MOX) and *N*-methyl-*N*-(tert-butyldimethylsilyl)-trifluoroacetamide + 1% tertbutyldime-thylchlorosilane (MTBSTFA + TBDMS) were commercially obtained from Thermo Scientific (Waltham, MA, USA).

### 2.2. Cell Culture and Gas Chromatography-Mass Spectrometry (GC-MS) Analysis

LCL and fibroblasts derived from two different families were cultured and tested as previously described [22,25]. In brief, LCL cells were maintained in RPMI 1640 supplemented with 15% FBS, P/S, and 2 mM of additional Gln. For experiments, 10 million LCL cells were incubated in T-25 flasks in 10 mL of complete RPMI (with dialyzed, dFBS) ± Asn for 24 h. At the end of the incubation, cell and media fractions were separated by centrifugation at 300× *g* for 5 min at 4 °C, and media was stored at −80 °C until subsequent processing and analysis. Cell pellets were thoroughly washed with 0.9% sterile saline, flash frozen, and stored at −80 °C. Patient-derived fibroblasts were maintained in DMEM supplemented with 10% FBS, ABAM (streptomycin, penicillin G, and amphotericin B), 1X NEAA, and 2 mM of additional Gln. For experiments, 1 million fibroblasts were plated in 100 mm cell culture dishes and incubated for 24 h to allow adherence, then DMEM ± Asn was added, and cells were incubated for an additional 24 h. At the end of the incubation period, media was collected and stored at −80 °C. Cells were harvested by trypsinization, washed with 0.9% saline, flash frozen, and stored at −80 °C. Cell and media samples were prepared and subjected to GC-MS analysis on a Thermo Scientific Single Quadrupole Mass Spectrometer (ISQ) and Gas Chromatograph (Trace 1310), exactly as reported previously [22,29,30,31].

### 2.3. Identification of Metabolites and Chromatogram Integration

The National Institute of Standards and Technology (NIST) mass spectrometry library and Xcalibur software (version 4.1) were used to identify metabolites within the cell and media samples [32]. A total ion chromatogram (TIC) was used to determine the peak areas of selected metabolites, which were then tabulated for statistical analysis. The Xcalibur software library batch processing method was utilized to integrate peak areas with ICIS peak fitting. Metabolite peak areas were further processed with Xcalibur Quan Browser to ensure proper peak fitting. All peak areas were normalized to the peak area of the internal standard, DL-norleucine. Targeted analysis of Asn by an external standard curve allowed for absolute quantification.

### 2.4. Statistical Analysis

Integrated peak intensities were imported into MetaboAnalyst (v5.0) and normalized by sum, followed by log_10_ transformation, and Pareto scaling. MetaboAnalyst was then utilized for principal component analysis (PCA), partial least square-discriminant analysis (PLS-DA), and hierarchical clustering analysis. Q^2^ and R^2^ values were determined by cross-validation to determine the fit of the PLS-DA model. Variable Importance in Projection (VIP) scores, representing the degree of influence of a variable, were leveraged to assess the importance of different metabolites to the PLS-DA model. Based on VIP scores, the top 25 compounds were plotted for each cell line and significant metabolites, established by Student’s *t*-test and FDR correction (α = 0.05), were used for Metabolite Set Enrichment Analysis (MSEA). Our statistical model was cross-validated in MetaboAnalyst (v5.0) by the leave-one-out method (LOOCV) as well as by receiver operating characteristic (ROC) curves to assess the predictive capability and the sensitivity/specificity of our model. LOOCV was performed across three components and ROC analysis was performed across all models with PLS-DA classification and feature ranking. Statistical significance across familial cells for both LCL and fibroblasts was established by one-way ANOVA with Tukey post-hoc analysis. Significant differences between cell lines as well as +Asn and −Asn media conditions for each cell line were established by two-way ANOVA with Šidák multiple comparisons correction and plotted to assess the direct effects of Asn deprivation on each individual metabolite pool.

## 3. Results

Two previous ASNSD studies utilized a lymphoblastoid immortalized cell line (LCL) model, a fibroblast model, and respective wild-type (WT) controls [22,25], which we have used for the present analysis. The LCLs are derived from a compound heterozygotic ASNSD patient with p.H205P and p.Y398Lfs*4 ASNS variants, as well as cells from the patient’s mother (p.H205P) and father (p.Y398Lfs*4). The cultured fibroblasts are derived from a different compound heterozygotic ASNSD patient with p.G373V and p.R519H ASNS variants, and the patient’s mother (p.R519H) and father (p.G373V). Each of these LCL and fibroblast cell lines have been previously characterized for *ASNS* mRNA expression, protein stability, and activity by in cellulo and in vitro assays [22,25].

### 3.1. GC-MS Analysis of Asparagine for Lymphoblastoid and Fibroblast Cell Lines

**Figure 1:**
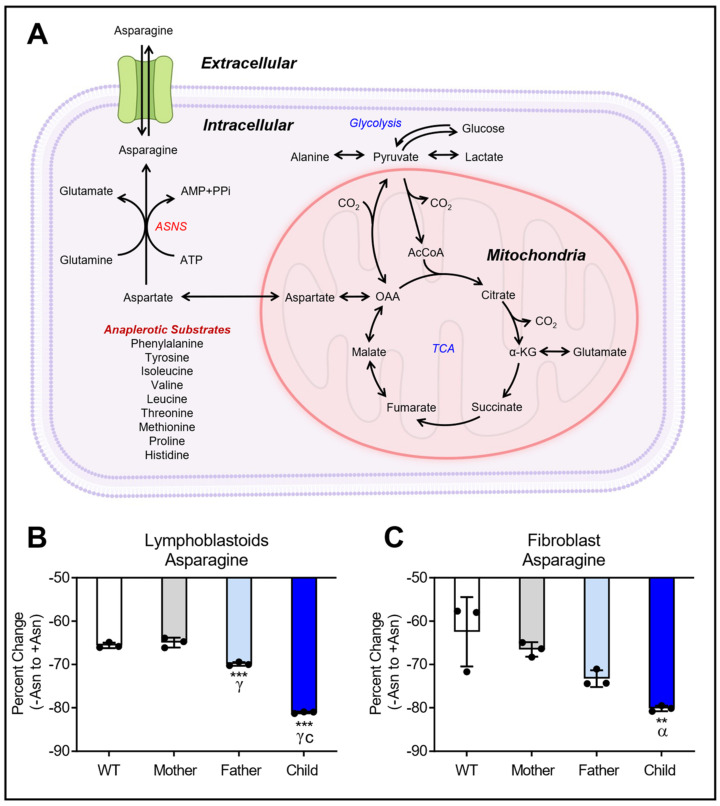
Intracellular analyses of asparagine (Asn) content of cells derived from two separate families carrying unique mutations in the *ASNS* gene. Panel (**A**) presents the ASNS reaction schematic of Asn production along with central metabolic pathways. Panel (**B**) shows the percent change of intracellular Asn measured in LCL cells generated from the first family. Panel (**C**) illustrates the percent change of intracellular Asn in the fibroblasts from the second family. Percent change is reported as the change of Asn content from −Asn to +Asn media conditions. Since Asn decreased in −Asn conditions, the percent change values are reported as negative. Intracellular Asn content was measured by targeted GC-MS analysis. All cell lines were tested as N = 3 biological replicates. The data are shown as the means ± SD. Statistical significance was established by ANOVA with Tukey post-hoc analysis and is represented as: (**) if *p* ≤ 0.01 and (***) if *p* ≤ 0.001 compared to WT. (α) if *p* ≤ 0.05 and (γ) if *p* ≤ 0.001 compared to Mother. (c) if *p* ≤ 0.001 compared to Father.

Intracellular Asn content was analyzed to assess the ability of LCL and fibroblast cell lines to generate Asn when challenged with Asn deprivation for 24 h. Following this incubation, no differences in cell numbers were observed between +Asn and −Asn media conditions. Figure 1A shows the general schematic of Asn production in mammalian cells. A targeted analysis of intracellular Asn was performed with an external standard curve for absolute quantification. The external standard curve covered 0 to 8000 ng/μL of Asn and showed excellent linearity of response with an R^2^ value greater than 0.99 (Supplementary Figure S1). Analysis of the percent change of Asn between +Asn and −Asn LCL showed significantly lower Asn content in the LCL from the ASNSD child compared to WT when deprived of Asn (Figure 1B). The father’s LCL showed a much smaller, but significant, decline in Asn, while there was no change in the mother’s Asn level. Across fibroblast cell lines, the child had significantly lower Asn content compared to the WT, as did the father to a lesser extent, when deprived of Asn (Figure 1C). Extracellular analysis of Asn content in both LCL and fibroblasts showed significantly lower Asn in all cells deprived of Asn with the lowest extracellular Asn among the family members occurring in the child’s cells (Supplementary Figures S3 and S4), which is consistent with previously reported data [22,25].

### 3.2. Analysis of Global Metabolomics

**Figure 2:**
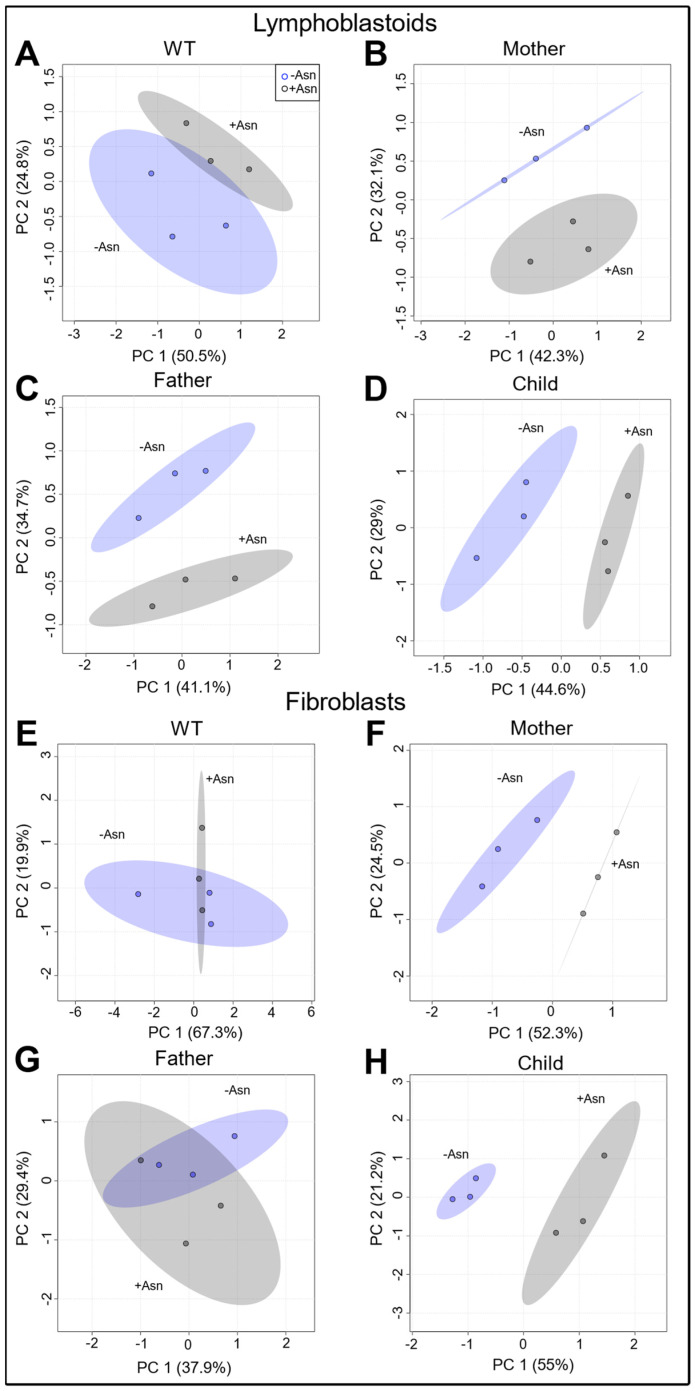
Principal component analysis of lymphoblastoids (LCL) and fibroblasts incubated with +Asn or −Asn media. All metabolites were normalized to DL-norleucine. Panels (**A**–**D**) represent 2D score plots for LCL and demonstrate evident separation between +Asn and −Asn conditions in maternal, paternal, and child cells with greater grouping between conditions in WT. 2D score plots for fibroblast cell lines demonstrate clear separation between conditions for maternal and child cells with greater evident grouping in WT and paternal cells (Panels **E**–**H**). For each 2D score plot, the shading indicates the 95% confidence intervals of each group.

A global overview of the metabolic profile of all cell lines under +Asn and −Asn media showed the strong effects of nutrient deprivation. Unsupervised principal component analysis (PCA) score plots were generated to assess the effects of Asn deprivation on the global metabolic profile of LCL and fibroblasts (Figure 2). As expected, WT LCL showed the lowest separation between +Asn and −Asn conditions (Figure 2A). Maternal, paternal, and child LCL showed evident group-dependent clustering and greater separation between +Asn and −Asn conditions than WT with PC1 percentages of 42.3%, 41.1%, and 44.6%, respectively (Figure 2B–D). Of note, maternal and paternal LCL appear to be more variable across PC2 (32.1% and 34.7%, respectively) than the child’s cells which are more variable across PC1. Similar trends are observed in the score plots of fibroblast cell lines (Figure 2E–H) which show minimal separation for WT and the father with PC1 percentages of 67.3% and 37.9%, respectively, greater separation in maternal cells (PC1 of 52.5%), and the greatest separation for the child’s cells across PC1 (55%).

**Figure 3:**
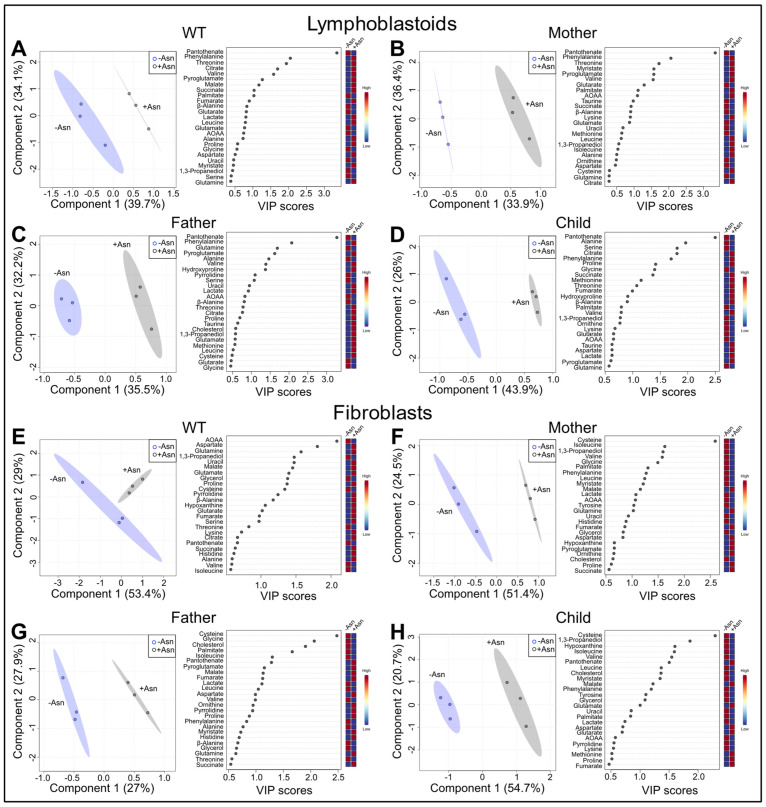
Supervised PLS-DA of LCL and fibroblasts incubated in medium with or without Asn. 2D score plots and VIP score plots are represented for LCL in panels (**A**–**D**) and fibroblasts in panels (**E**–**H**). The percentage of variance for components 1 and 2 are represented in the axes of each PLS-DA 2D score plot. For each 2D score plot, the shading indicates the 95% confidence intervals for each group. The VIP score plots of the top 25 metabolites were derived from the PLS-DA analysis and represent the distinct differences between cells incubated in media with or without Asn.

To further analyze the data, we performed supervised PLS-DA (Figure 3). PLS-DA 2D score plots are presented in Figure 3A–D for LCL and Figure 3E–H for fibroblasts. All LCL showed exemplary R^2^ and Q^2^ values from leave-one-out cross validation (Supplementary Figure S5) with R^2^ values approximating unity, indicating strong certainty as well as requisite predictive accuracy inclusive of three components of the model. Receiver operating characteristic (ROC) curve analysis showed that our model has high specificity and sensitivity for all cell lines in LCL as well as mother, father, and child fibroblasts (Supplementary Figure S6). The 2D score plots of maternal and child fibroblasts showed strong separation between +Asn and −Asn media conditions. Paternal fibroblasts showed a lower PC1 of 27% as well as lower Q^2^ values, but the WT fibroblasts failed cross-validation which is consistent with ROC results showing that our model has lower specificity and sensitivity for WT fibroblasts (Supplementary Figures S5 and S6). Overall, each of the LCL showed a clear separation after incubation in +Asn and −Asn media conditions, whereas the fibroblasts only showed robust separation of the three cell lines from the ASNSD family members.

From the PLS-DA method, we assessed the VIP scores of the top 25 metabolites and observed distinct alterations in the metabolic profile of all cell lines, with the exception of WT fibroblasts, as a function of Asn deprivation. In all LCL, pantothenate, or vitamin B5, was shown to increase and had the greatest VIP score (>2.5) among the top 25 metabolites. Comparing WT and child LCL +/−Asn, the cell lines shared 80% of altered metabolites, indicating similar pathways being affected by Asn deprivation regardless of ASNS activity level (Figure 3A,D). Interestingly, all four LCL lineages, after incubation in an Asn-deficient medium, showed decreases in glycolytic products, TCA cycle intermediates, and anaplerotic substrates. In contrast, based on VIP score analysis, after the child’s fibroblasts were deprived of Asn they exclusively exhibited decreases in TCA cycle intermediates and anaplerotic substrates such as methionine and proline (Figure 3H). Additionally, cysteine was found to be elevated and had the greatest VIP score in maternal, paternal, and child cells (Figure 3E–H). Maternal and paternal fibroblast cells showed similar trends to the child’s cells (Figure 3F,G).

To assess the effects of Asn deprivation on specific metabolic pathways, we performed Metabolite Set Enrichment Analysis (MSEA) on the significantly different (*p* ≤ 0.05) metabolites between +Asn and −Asn groups for child LCL and fibroblasts (Supplementary Figure S2). MSEA of child LCL showed enriched glutathione metabolism as well as enrichment in glycolytic, anaplerotic, and TCA cycle pathways (Supplementary Figure S2). Similar to the LCL lineages, fibroblasts derived from the child also exhibited enrichment in glutathione metabolism as well as anaplerotic and TCA cycle pathways (Supplementary Figure S2).

### 3.3. Hierarchical Clustering Analysis

**Figure 4:**
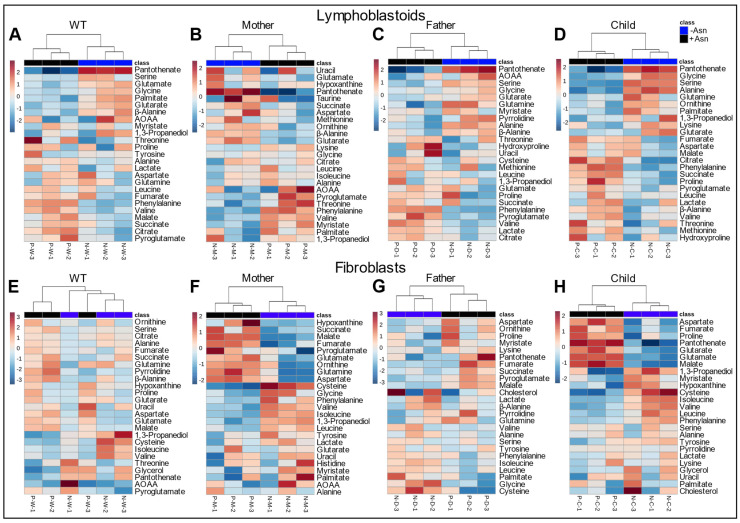
Hierarchical clustering between +Asn and −Asn LCL (**A**–**D**) and fibroblasts (**E**–**H**). Heatmaps discern differential metabolite levels across the top 25 metabolites determined by analysis between cells incubated in +Asn and −Asn medium. Statistical significance was identified by *p* ≤ 0.05.

To further assess the variability in the global metabolomics data, hierarchical clustering was performed to generate heat maps of the top 25 metabolites that were significantly altered (Figure 4). The analysis shows clear clustering of all individual replicates into +Asn and −Asn groups for both LCL (Figure 4A–D) and fibroblasts (Figure 4E–H). Similar to PLS-DA and VIP score analysis, hierarchical clustering showed a decrease in TCA cycle intermediates as well as anaplerotic substrates for all LCL deprived of Asn. With regard to the fibroblast cell lines, the WT showed greater variability between +Asn and −Asn conditions when compared to all three of the ASNSD-derived cell lines, which coincides with the grouping observed in the PCA and PLS-DA analysis. Conversely, across the family-derived cells, decreases in TCA cycle intermediates were observed.

### 3.4. Metabolites Identified in Common

**Figure 5:**
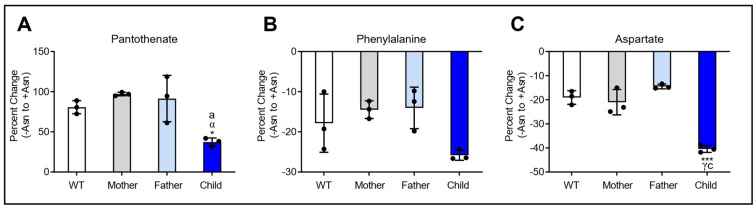
Signature metabolic differences found across LCL and fibroblasts challenged with Asn deprivation. Data are presented as the percent change between +Asn and −Asn media conditions for each cell line. Pantothenate (**A**) and phenylalanine (**B**) were both altered in all LCL lineages, and aspartate (**C**) was decreased in all fibroblast cell lines. The data are shown as means ± SD and statistical significance was established by ANOVA with Tukey post-hoc analysis and is represented as: (*) if *p* ≤ 0.05 and (***) if *p* ≤ 0.001 compared to WT. (α) if *p* ≤ 0.05 and (γ) if *p* ≤ 0.001 compared to Mother. (a) if *p* ≤ 0.05 and (c) if *p* ≤ 0.001 compared to Father.

To identify potential biomarkers of Asn deprivation, metabolites were analyzed by PLS-DA, VIP scores, and Student’s *t*-test between +Asn and −Asn conditions for each cell line. Of the top 25 significantly altered metabolites identified by these criteria, pantothenate and phenylalanine were found to be universally changed between +Asn and −Asn conditions across all LCL lineages, whereas a change in aspartate was in common across all fibroblast cell lines (Figure 5). All three of these metabolites were plotted as a function of percent change between +Asn and −Asn conditions, and they exhibited similar behaviors for WT, maternal, paternal, and child cells for their respective cell line model. In LCL, the child-derived cells had significantly lower pantothenate compared to WT and maternal cells when challenged with Asn deprivation (Figure 5A). Similarly, the child’s LCL had significantly lower phenylalanine compared to maternal cells (Figure 5B). For the fibroblast lines, the child’s cells had significantly lower aspartate compared to the WT or either parent (Figure 5C).

### 3.5. Asparagine Deprivation Significantly Perturbs Central Carbon Metabolism

**Figure 6:**
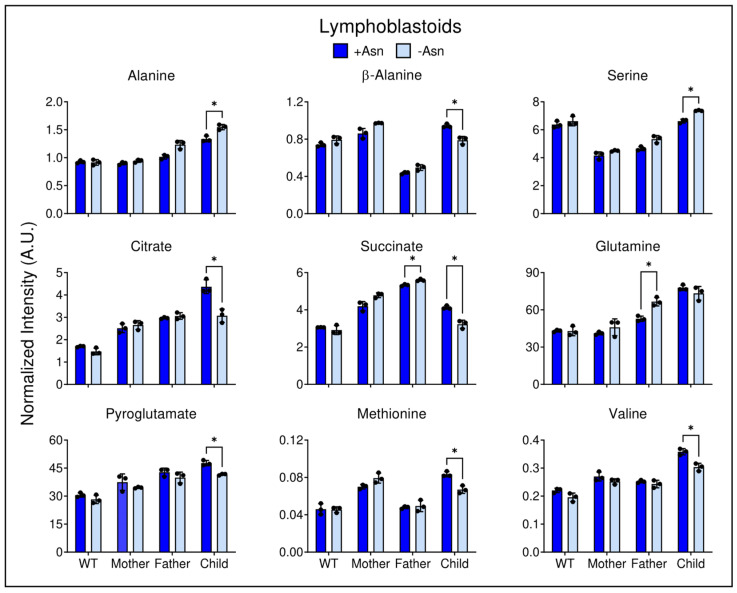
Differential metabolite levels between +Asn and −Asn groups in LCL. Asn deprivation in ASNS-deficient cells leads to significant decrements in TCA cycle intermediates, anaplerotic substrates, and amino acids. All cell lines were tested as N = 3 biological replicates. The data are shown as means ± SD and statistical significance was established by two-way ANOVA analysis with Šidák multiple comparisons correction, represented as: (*) if *p* ≤ 0.05 compared between +Asn and −Asn groups for each cell line.

**Figure 7:**
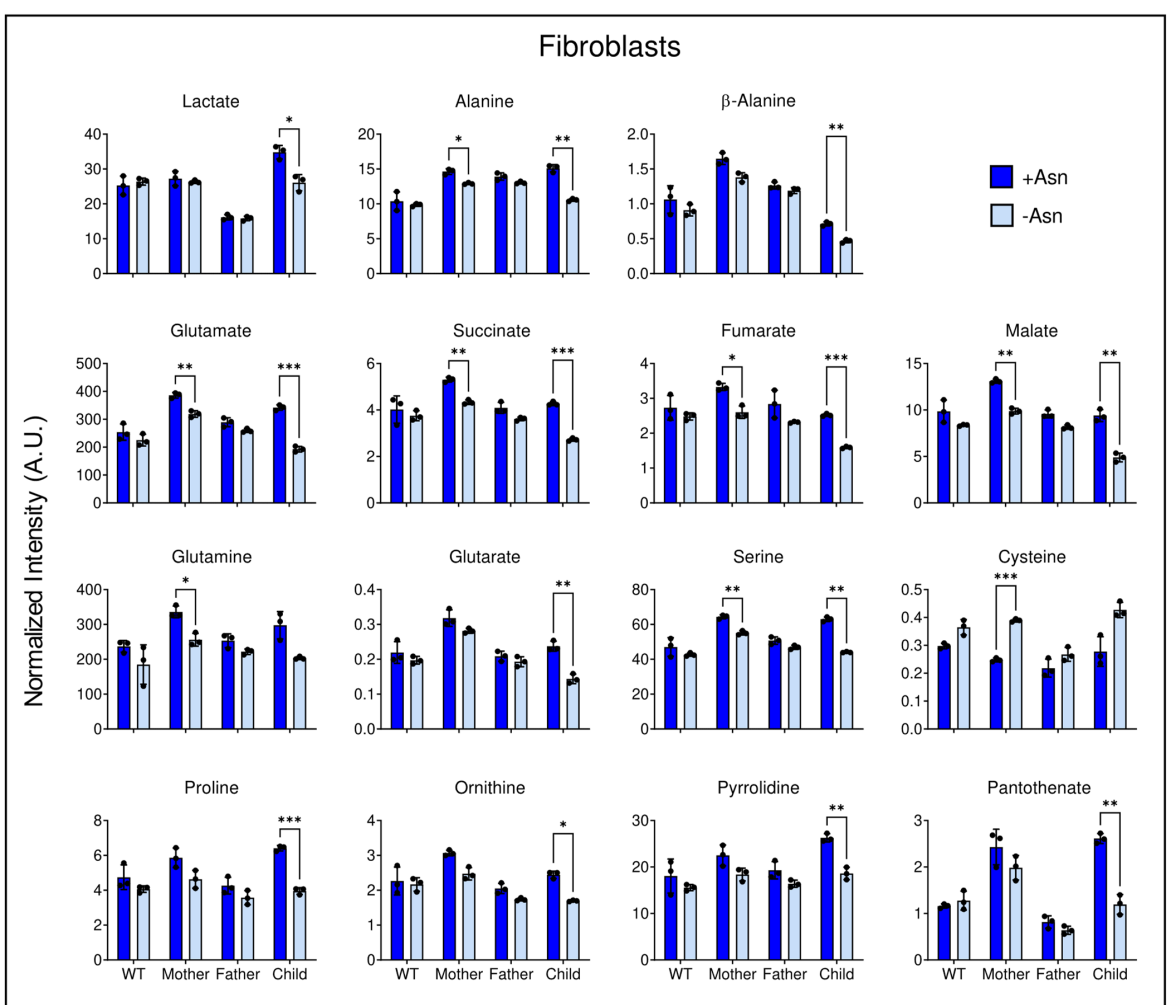
Differential metabolite levels between +Asn and −Asn groups in fibroblasts. Asn deprivation in ASNS-deficient cells leads to significant decrements in glycolytic products, TCA cycle intermediates, anaplerotic substrates, and amino acids. All cell lines were tested as N = 3 biological replicates and the data are shown as means ± SD. Statistical significance was established by two-way ANOVA analysis with Šidák multiple comparisons correction and is represented as: (*) if *p* ≤ 0.05, (**) if *p* ≤ 0.01, and (***) if *p* ≤ 0.001 compared between +Asn and −Asn groups for each cell line.

To directly assess significantly different metabolites, the normalized intensity levels of metabolites were analyzed between +/−Asn conditions for both LCL (Figure 6) and fibroblast cell lines (Figure 7). Metabolites from Supplementary Table S1 were analyzed based on two-way ANOVA with multiple comparisons correction statistical significance (*p* ≤ 0.05) between +Asn and −Asn conditions for each metabolite and each cell line, independently. Figure 6 demonstrates that for WT LCL challenged with Asn deprivation, only pantothenate and phenylalanine showed differences (Figure 5). In contrast, the child’s LCL deprived of Asn showed significant decrements in TCA cycle intermediates, as well as branched-chain amino acids and anaplerotic substrates such as valine and methionine. Conversely, the child’s LCL showed significant increases in alanine and serine, a result similar to that observed in the paternal LCL lineage challenged with Asn deprivation (Figure 6). In contrast to the child-derived LCL, the maternal and paternal cell lines showed significant increases in some TCA cycle intermediates as well as in glutamine (Figure 6).

WT fibroblast cells showed a significant decrease in aspartate (Figure 5) and an increase in cysteine (Figure 7). It is noted that the child-derived LCL and fibroblasts exhibited similar trends. Specifically, deprivation of medium Asn lead to lower levels of glycolytic products, TCA cycle intermediates, anaplerotic substrates, and amino acids. Although not to the same quantitative magnitude, similar trends were observed in the maternally-derived fibroblasts. These changes occurred to an even lesser extent in the paternal fibroblast cells (Figure 7).

## 4. Discussion

Since the discovery of asparaginase as an anti-cancer agent for treating acute lymphoblastic leukemia, ASNS and Asn deprivation have been important areas of research [15,16]. Several studies have observed a positive correlation between ASNS expression and tumor growth [3,10,11,12,33,34,35]. Additionally, recent research on ASNSD has extensively probed the molecular consequences of Asn deprivation on patient cells deficient of ASNS and found that cell survival correlates with Asn supplementation. It has been established that Asn is required to sustain protein synthesis and cell growth in both cancer and healthy cells [36,37,38,39]. There have been several metabolic analyses testing the implications of ASNS in cancer, but there is limited knowledge about the effects of Asn deprivation on the global metabolomics of the inborn error of metabolism, ASNSD. Based on a review of the literature, this is the first global metabolomic analysis assessing the metabolic profile changes of healthy and ASNSD cells challenged with Asn deprivation.

PCA analysis demonstrated changes in the global metabolic profile between cells supplemented with Asn and cells deprived of Asn. As expected, the extent of variability in the principal components increased in ASNS-deficient LCL and fibroblast cells derived from ASNSD patients, while minimal separation was observed for the +Asn and −Asn groups of WT controls (Figure 2). This result indicates that, even in the global metabolic context, ASNS activity has a fundamental effect on the metabolic status of the cell, beyond protein synthesis. PLS-DA, the derived VIP scores, and hierarchical analysis for both LCL and fibroblasts, identified many common metabolites between the groups that were the underlying factors driving the variability detected by the PCA analysis (Figure 3 and Figure 4). Hierarchical clustering analysis was unsupervised, highlighting the consistency of our data and the power of our analysis. Our cross-validation and ROC analysis demonstrate that the results observed from our statistical models accurately represent metabolic changes induced by Asn deprivation with high sensitivity and specificity, with the exception of WT fibroblasts, which failed cross-validation. We found pantothenate and phenylalanine as potential metabolic signatures of Asn deprivation in healthy and diseased LCL, and we identified aspartate as a potential signature in familial fibroblasts (Figure 5). Pantothenate and aspartate were both significantly decreased across ASNS-deficient patient cells (Figure 5). These results need to be further explored as previous efforts to analyze Asn content in blood plasma found that Asn was low in only about half of the ASNSD patients assessed, and that measuring Asn plasma content is not sensitive enough for ASNSD disease diagnosis [40].

A deeper analysis of the most significantly changed metabolites demonstrated that Asn deprivation ultimately leads to a breakdown of central carbon metabolism. Whether or not these changes are associated with a reduction in protein synthesis in ASNS deficient cells remains to be established. Fibroblast cells derived from ASNSD children showed significant decreases in intracellular lactate levels upon Asn deprivation, indicating a possible reduction in glycolytic activity (Figure 7). When the fibroblasts from the ASNSD child were deprived of Asn, there was a reduction in alanine, suggesting that glucose may be preferentially routed as anaplerotic pyruvate to help compensate for reductions in TCA cycle intermediates. Lactate to alanine ratios indicate a significant cytosolic NADH/NAD^+^ perturbation in Asn deprived ASNSD LCL, whereas no difference between media conditions was observed in ASNSD fibroblasts (Supplementary Figure S7). LCL ASNSD cells challenged with Asn deprivation exhibited significant decline in the levels of citrate and succinate (Figure 6), and ASNSD fibroblasts displayed declines in aspartate, glutamate, succinate, fumarate, and malate, indicating dysregulated anaplerosis or cataplerosis (Figure 5, Figure 6 and Figure 7). This decrement in TCA cycle intermediate pool sizes is matched by significant reductions in anaplerotic substrates observed in both LCL and fibroblasts, which indicates that ASNS-deficient cells may be actively consuming intracellular phenylalanine, methionine, tyrosine, proline, histidine, and branched-chain amino acids in an attempt to maintain cellular homeostasis either in energy metabolism or in protein turnover. These data are consistent with results in the child-derived LCL deprived of Asn, which demonstrated a significant reduction in extracellular valine, leucine, and isoleucine compared to cells incubated in +Asn media (Supplementary Figure S3).

The systemic changes observed in individual metabolite levels between +Asn and −Asn conditions in ASNS deficient cells are matched by MSEA, which shows pathway enrichments in redox, anaplerotic, and TCA cycle pathways for both LCL and fibroblasts (Supplementary Figure S2). The Asn deprivation-dependent impairments we observed in aspartate, TCA cycle intermediates, and redox balance metabolites such as glutamate, serine, and cysteine are in agreeance with metabolic dysfunction in Asn-starved sarcoma cells, as previously reported [39]. This indicates that, to some degree, ASNS deficiency in ASNSD and cancer is culminating in similar metabolic downfalls.

While a primary function of Asn is to sustain protein synthesis, recent reports have shown that Asn can also serve as an amino acid exchange factor to promote cancer cell growth [11]. Krall et. al. (2016) proposed that intracellular Asn was exchanging with extracellular amino acids such as serine. To assess this novel role of Asn in our cell culture models we performed GC-MS-based extracellular metabolite panels (Supplementary Figures S3 and S4). Intriguingly, we observed significantly higher extracellular serine in WT, paternal, and child LCL deprived of Asn, as well as slightly higher extracellular serine for maternal cells (Supplementary Figure S3). These data suggest that reduced intracellular Asn may lead to a decrease in exchange potential for extracellular serine in healthy and ASNSD affected LCL, which is the same observation previously reported in cancer cells. However, this effect was not observed in ASNSD fibroblast cells which indicates that it may be tissue or cell-type specific. Given that a decrease in exchange was observed in Asn-deprived WT cells, we suggest that Asn as an exchange factor plays a secondary role in promoting cell survival in this model.

In summary, our findings demonstrate that Asn deprivation leads to a broad-based disruption of the metabolic profiles of patient cells deficient for ASNS. We have identified potential metabolic signatures of Asn deprivation in healthy and ASNSD cells. This observation is of high interest for ASNSD and metabolic research as it warrants future work to establish the relevance of these metabolic signatures and their potential roles as diagnostic and prognostic biomarkers in an in vivo and clinical setting. While our results indicate a certain global metabolic insult caused by Asn deprivation in ASNS deficient cells, further work must be done to discern pathway-specific changes using isotopomer tracer and metabolic flux analysis. The research reported here warrants future studies on ASNSD cell models to assess the utilization of [U-^13^C]glucose to analyze central carbon metabolism, [U-^13^C]aspartate to assess the specific fate of the ASNS reaction, and [U-^13^C]glutamine to assess TCA cycle turnover. Additionally, the significant decrements in lactate and central metabolism suggest that in vivo [^2^H_7_]glucose metabolic imaging may potentially be utilized to assess the effects of Asn deprivation on brain metabolism [41].

## Data Availability

The data presented here are available through this article, Supplementary Materials, or upon request. Please contact the authors for more information.

## Supplementary Materials

The following supporting information can be downloaded at: https://www.mdpi.com/article/10.3390/nu15081938/s1, Figure S1: Linearity of response for the Asn external calibration curve in the targeted GC-MS analysis.; Figure S2: Metabolite Set Enrichment Analysis (MSEA) was performed on child lymphoblastoid (LCL) and fibroblast cell lines; Figure S3: Differential extracellular metabolite levels lymphoblastoid cell lines (LCL); Figure S4: Differential extracellular metabolite levels for fibroblasts; Figure S5: PLS-DA cross validation plots for LCL and fibroblast cell lines; Figure S6: Receiver operating characteristic (ROC) curves plotted for LCL and fibroblast cell lines; Figure S7: Lactate to alanine ratios in LCL and fibroblast cell lines incubated in medium +Asn and −Asn; Table S1: m/z ions of analyzed metabolites.
