# Supplementary material for: Metabolomic Profiling of Asparagine Deprivation in Asparagine Synthetase Deficiency Patient-Derived Cells
Source: Nutrients. 2023 Apr 18;15(8):1938. doi: 10.3390/nu15081938 (PMC10145675; doi:10.3390/nu15081938)
Supplement: Supplementary file 1 [file nutrients-15-01938-s001.zip › nutrients-2270050-supplementary.pdf]

nutrients

Article

Metabolomic Profiling of Asparagine Deprivation in Asparagine Synthetase Deficiency Patient-Derived Cells

Mario C. Chang [1], Stephen J. Staklinski [1,2], Vinay R. Malut [1], Geraldine L. Pierre [1], Michael S. Kilberg [1] and Matthew E. Merritt [1,*]

[1] Department of Biochemistry and Molecular Biology, University of Florida College of Medicine, Gainesville, FL 32610, USA
[2] School of Biological Sciences, Cold Spring Harbor Laboratory, Cold Spring Harbor, NY 11724, USA
* Correspondence: matthewmerritt@ufl.edu

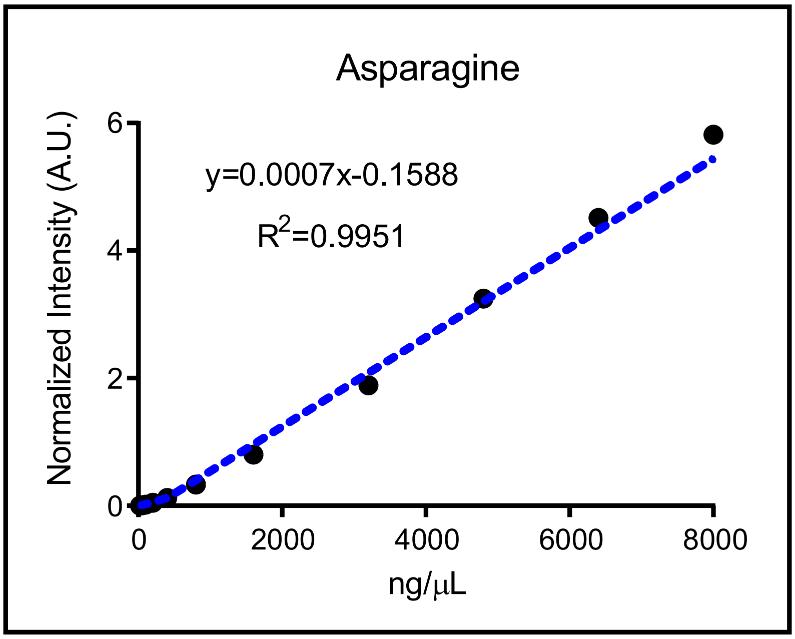

Figure S1. Linearity of response for the Asn external calibration curve in the targeted GC-MS analysis.

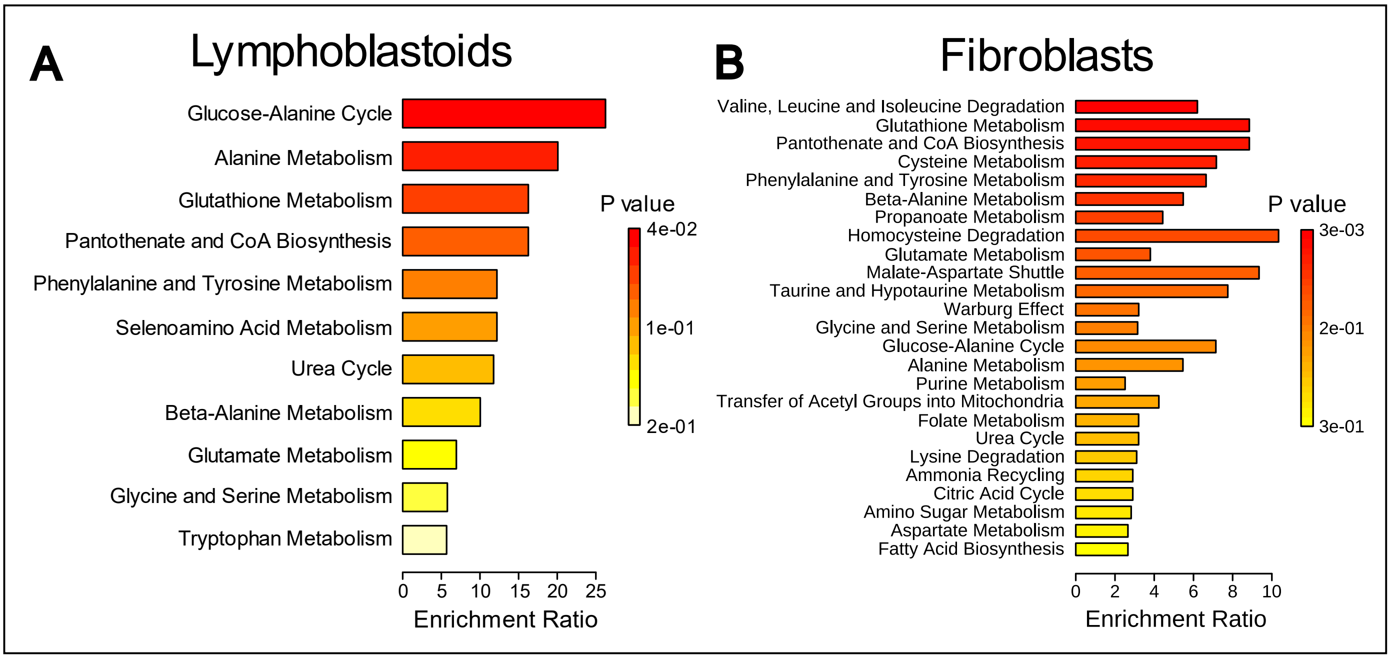

Figure S2. Metabolite Set Enrichment Analysis (MSEA) was performed on child lymphoblastoid (LCL) and fibroblast cell lines. The 25 most significantly altered metabolites, established by Student's *t*-test and FDR correction (α=0.05), between +Asn and -Asn conditions were assessed. MSEA allows for the investigation of enriched metabolic pathways based on the significant enrichment of functionally or metabolically related metabolites.

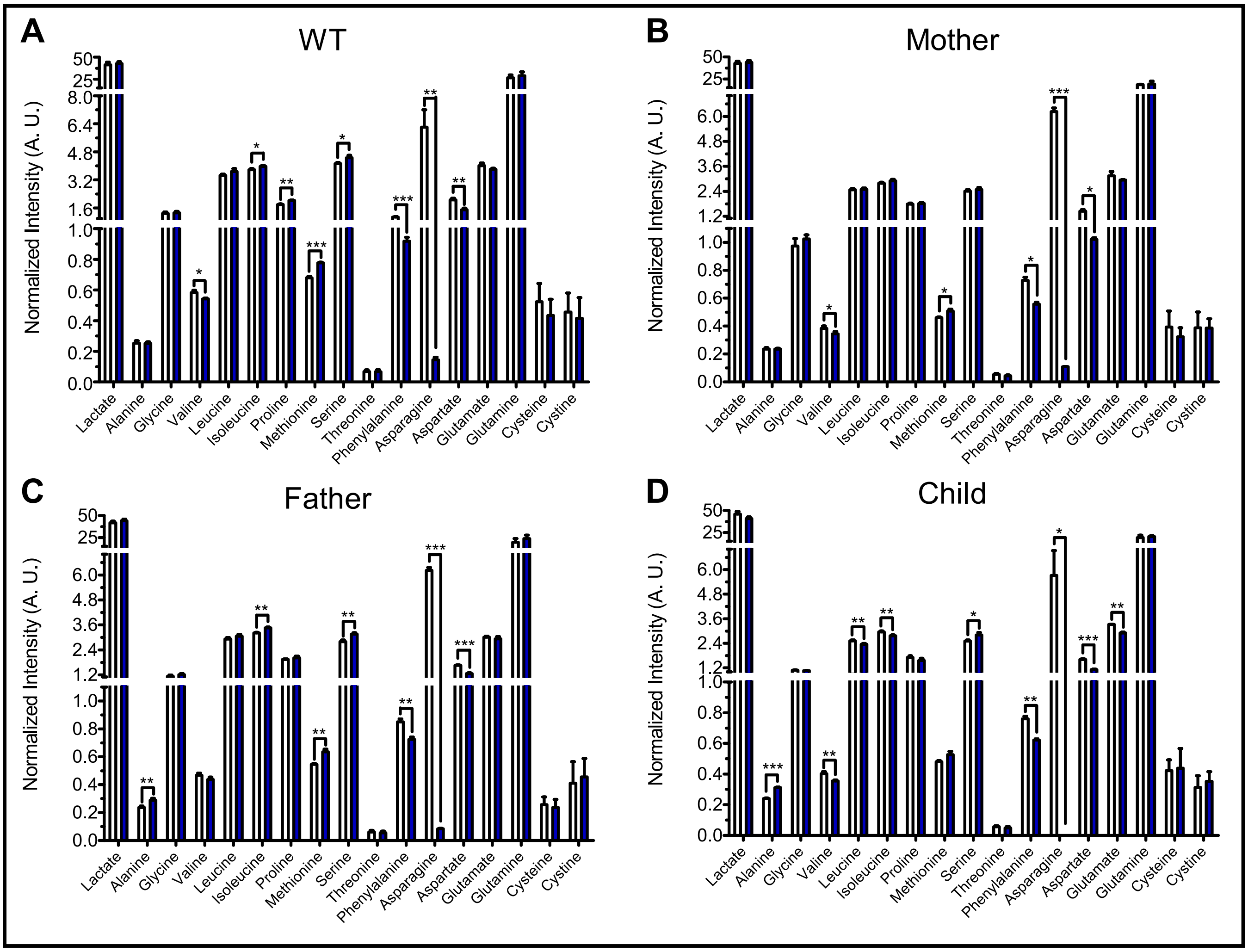

Figure S3. Differential extracellular metabolite levels lymphoblastoid cell lines (LCL). WT, maternal, paternal, and child LCL were incubated in medium +Asn (White Bars) and -Asn (Blue Bars). All cell lines were tested as N=3 biological replicates. The data is shown as means ± SD and statistical significance was established by Student's *t*-test analysis and is represented as: (*) if $P \leq 0.05$, (**) if $P \leq 0.01$, and (***) if $P \leq 0.001$ compared between +Asn and -Asn groups for each cell line.

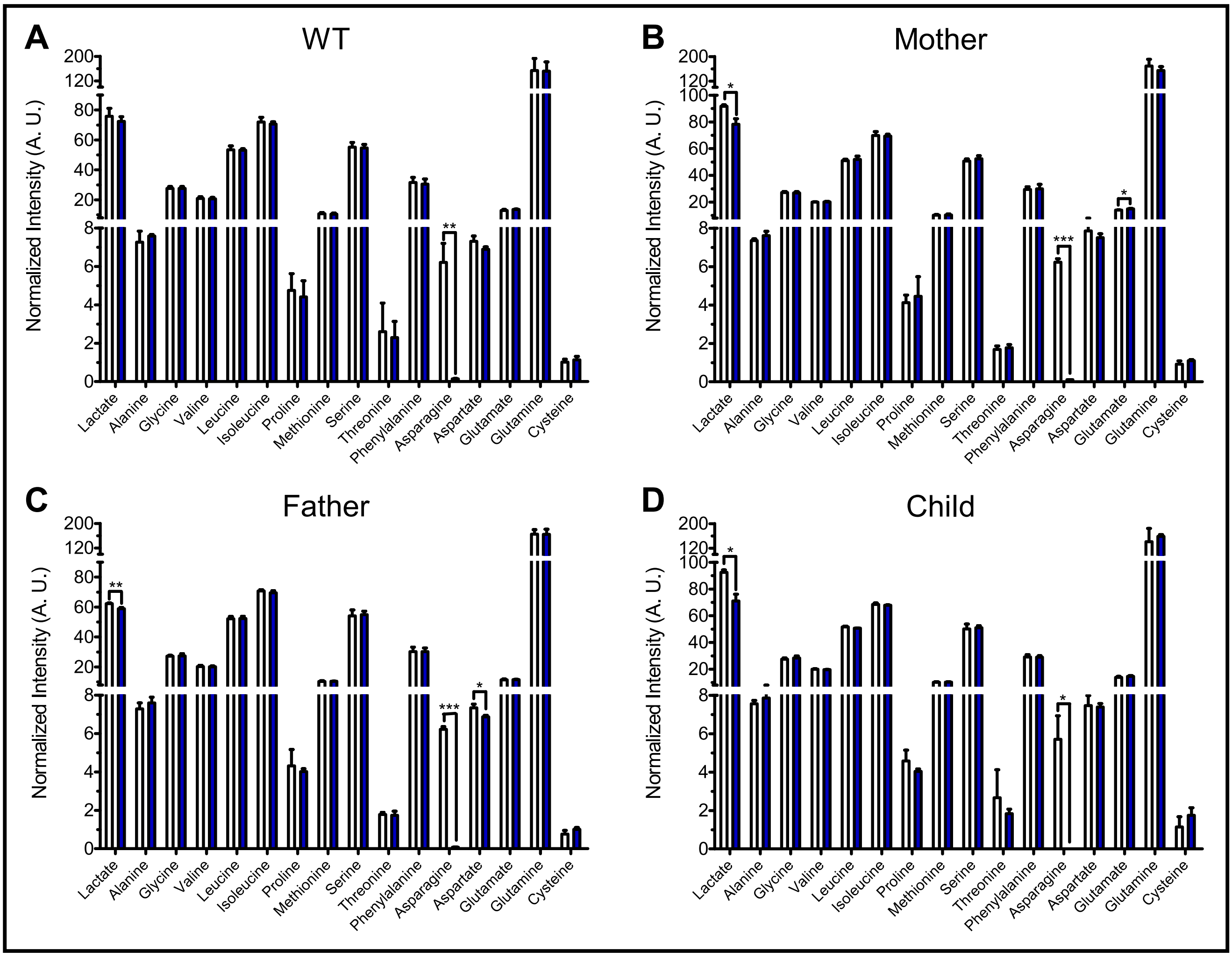

Figure S4. Differential extracellular metabolite levels for fibroblasts. WT, maternal, paternal, and child fibroblasts were incubated in medium +Asn (White Bars) and -Asn (Blue Bars). All cell lines were tested as N=3 biological replicates. The data are shown as means ± SD and statistical significance was established by Student's *t*-test analysis and is represented as: (*) if $P \leq 0.05$, (**) if $P \leq 0.01$, and (***) if $P \leq 0.001$ compared between +Asn and -Asn groups for each cell line.

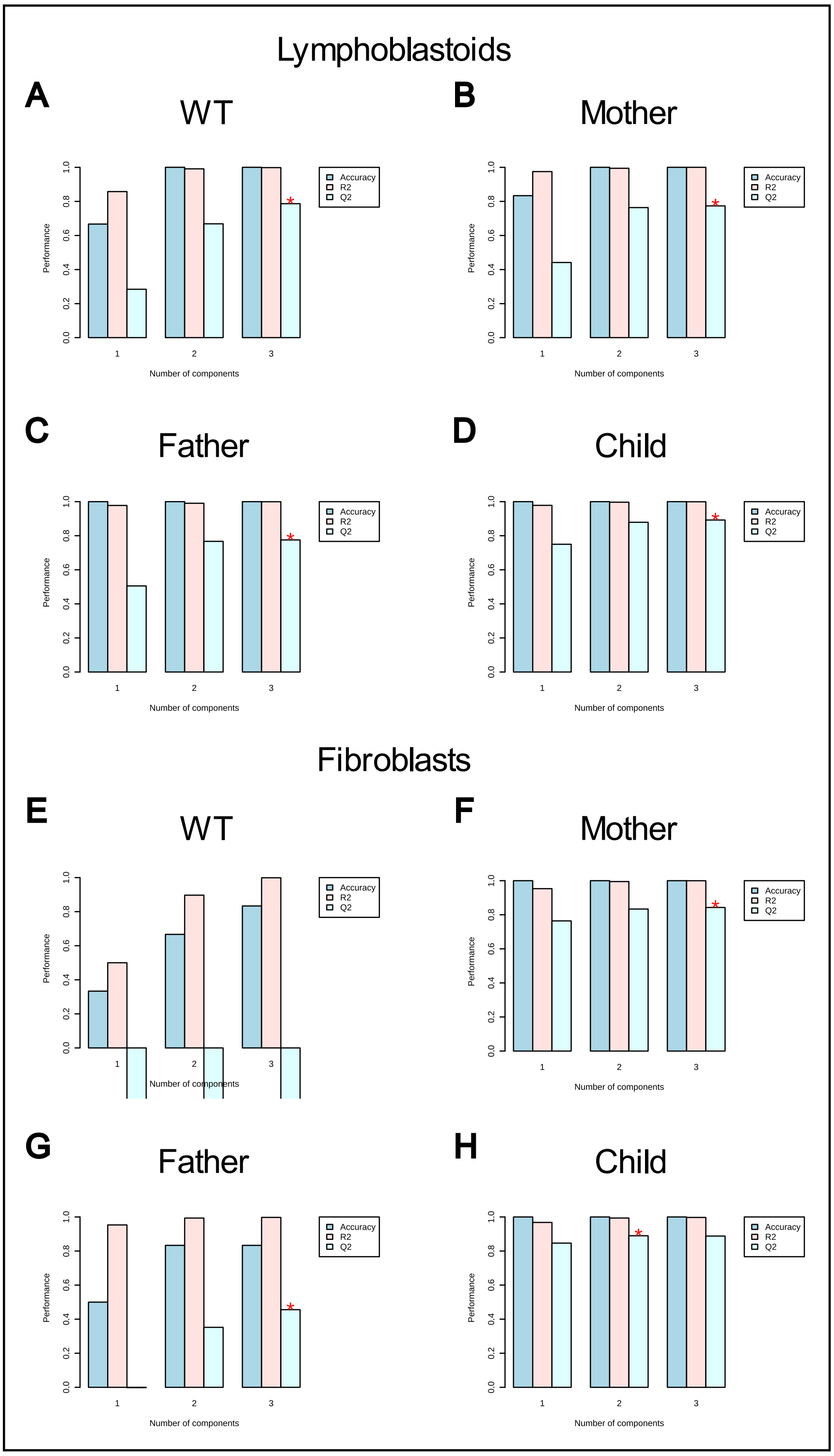

Figure S5. PLS-DA cross validation plots for LCL and fibroblast cell lines. Data is presented for WT, Mother, Father, and Child cells.

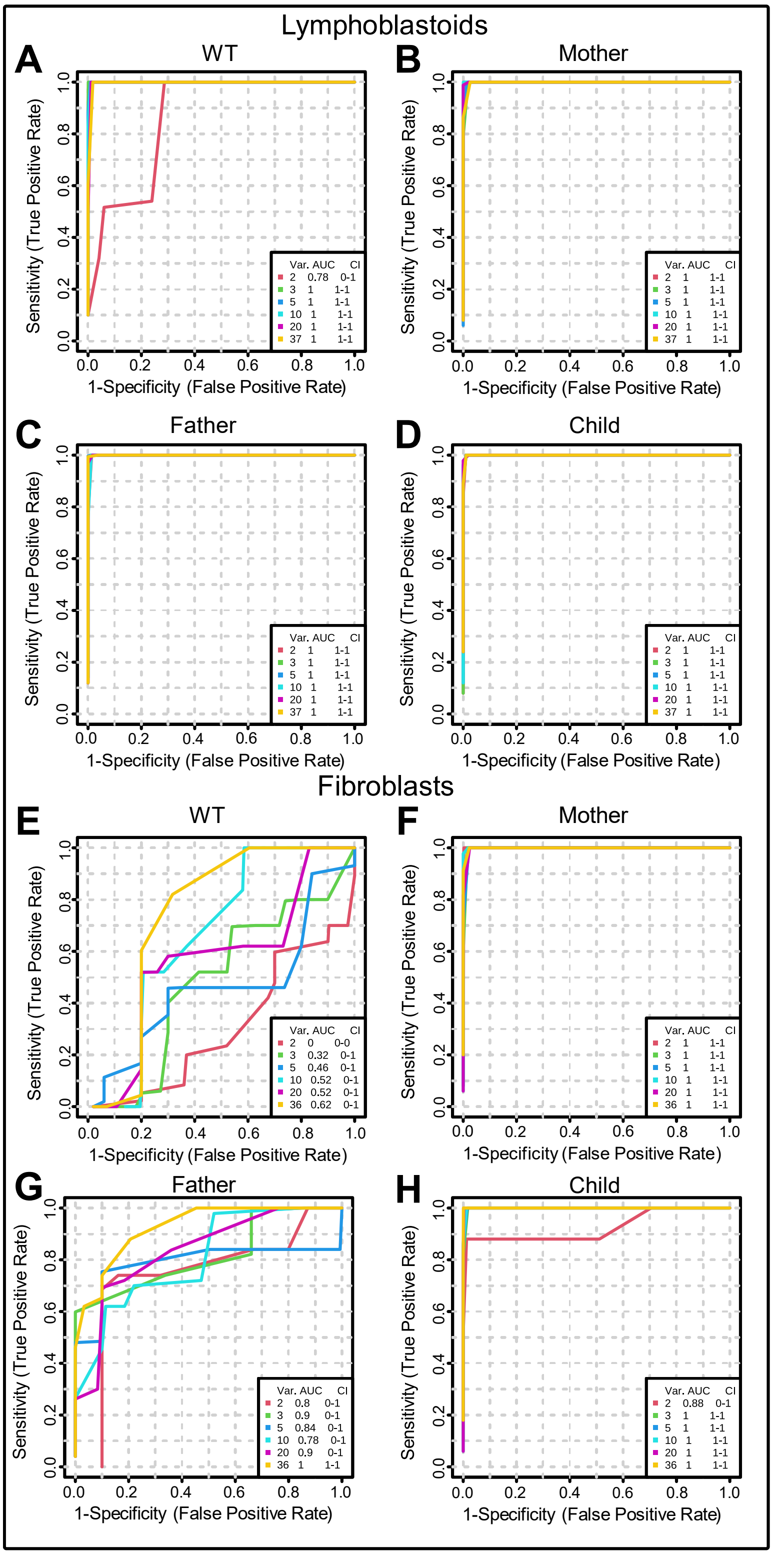

Figure S6. Receiver operating characteristic (ROC) curves plotted for LCL and fibroblast cell lines. Data is presented for WT, Mother, Father, and Child cells.

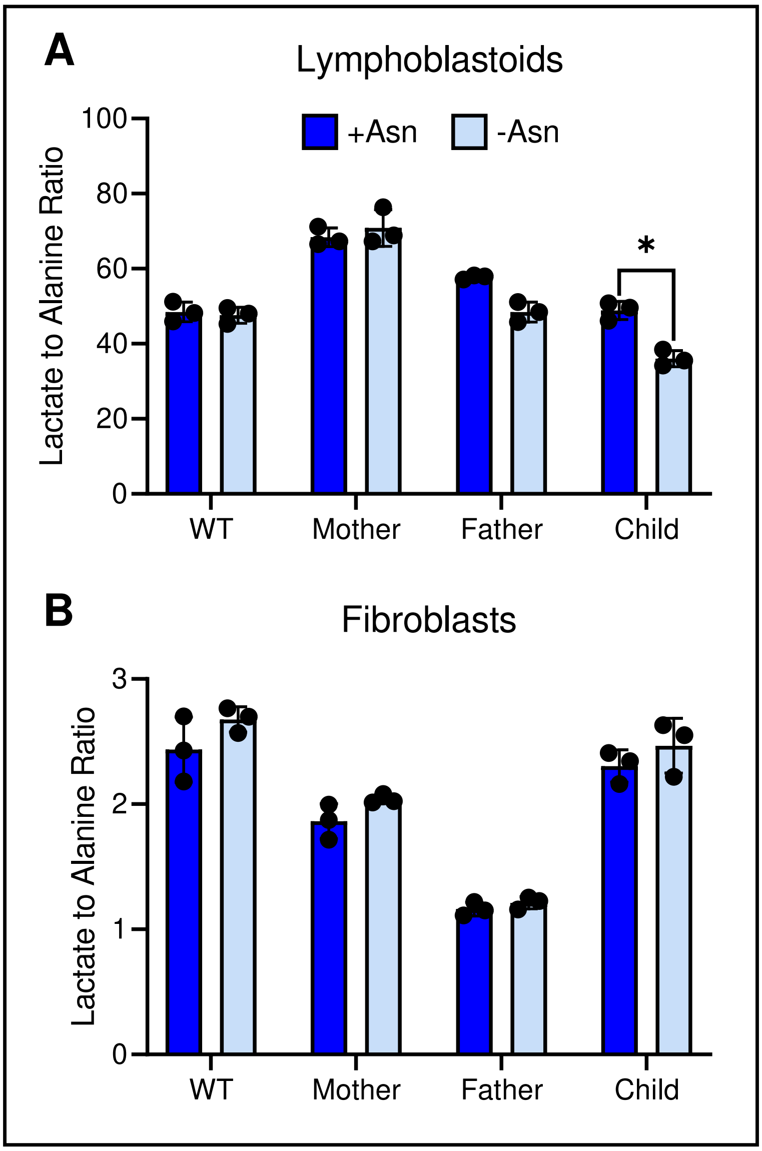

Figure S7. Lactate to alanine ratios in LCL and fibroblast cell lines incubated in medium +Asn and -Asn. The data are shown as means ± SD and statistical significance was established by two-way ANOVA analysis with Šidák multiple comparisons correction and is represented as: (*) if $P \leq 0.05$ compared between +Asn and -Asn groups for each cell line.

Table S1. m/z ions of analyzed metabolites

Analyte	m/z
Lactate	261
Alanine	260
Glycine	246
Valine	302
Leucine	302
Isoleucine	302
Norleucine	200
Succinate	289
Proline	258
Fumarate	287
Glycerol	377
Pyroglutamine	300
Methionine	320
Serine	390
Threonine	417
Myristic Acid	285
Phenylalanine	308
Malate	419
Aspartate	418
Glutarate	433
Glutamate	432
Asparagine	417
Palmitic Acid	313
Ornithine	286
Lysine	300
Glutamine	431
Oleic Acid	339
Stearic Acid	341
Histidine	440
Citrate	591
Cystine	348
Cholesterol	367
1,3-Propanediol	247
Aminooxyacetic Acid	262
Uracil	283
Taurine	296
Pyrrolidine-1,2-dicarboxylic Acid	330
Hydroxyproline	416
Cysteine	406
Hypoxanthine	307
Tyrosine	466
Pantothenic Acid	504
β-Alanine	218
